# Rapid Prototyping of Polymeric Nanopillars by 3D Direct Laser Writing for Controlling Cell Behavior

**DOI:** 10.1038/s41598-017-09208-y

**Published:** 2017-08-23

**Authors:** Nina Buch-Månson, Arnaud Spangenberg, Laura Piedad Chia Gomez, Jean-Pierre Malval, Olivier Soppera, Karen L. Martinez

**Affiliations:** 1 Bionanotechnology and Nanomedicine Laboratory, Department of Chemistry and Nano-science Center, University of Copenhagen, Universitetsparken 5, DK-2100 Copenhagen, Denmark; 2 Institut de Science des Matériaux de Mulhouse (IS2M), CNRS - UMR 7361, Université de Haute Alsace, 15 rue Jean Starcky, Mulhouse, France

## Abstract

Mammalian cells have been widely shown to respond to nano- and microtopography that mimics the extracellular matrix. Synthetic nano- and micron-sized structures are therefore of great interest in the field of tissue engineering, where polymers are particularly attractive due to excellent biocompatibility and versatile fabrication methods. Ordered arrays of polymeric pillars provide a controlled topographical environment to study and manipulate cells, but processing methods are typically either optimized for the nano- or microscale. Here, we demonstrate polymeric nanopillar (NP) fabrication using 3D direct laser writing (3D DLW), which offers a rapid prototyping across both size regimes. The NPs are interfaced with NIH3T3 cells and the effect of tuning geometrical parameters of the NP array is investigated. Cells are found to adhere on a wide range of geometries, but the interface depends on NP density and length. The Cell Interface with Nanostructure Arrays (CINA) model is successfully extended to predict the type of interface formed on different NP geometries, which is found to correlate with the efficiency of cell alignment along the NPs. The combination of the CINA model with the highly versatile 3D DLW fabrication thus holds the promise of improved design of polymeric NP arrays for controlling cell growth.

## Introduction

Nano- and microtopography mimicking the environment of the extracellular matrix has been widely employed for *in vitro* studies of cell behavior with the prospect of designing better implants and engineering tissue^1, 2^. Surface features on the nano- and microscale have been obtained through the shaping of a wide variety of materials^2, 3^, but polymers are particularly convenient due to low-cost and versatile fabrication methods^4^. Furthermore, polymers have an excellent biocompatibility and some are even biodegradable, which is of utmost importance in the context of implant technology^5^. Another attractive feature is optical transparency, which eases the imaging analysis of cells on or inside polymeric structures. The versatility of polymer materials is reflected in the numerous cell studies on a variety of polymeric structures, such as lines or gratings^6–8^, nanopores^9, 10^, and square^11–13^, triangular^14^, round^6, 15–17^ or even bridged^18^ pillars. Among these, ordered arrays of vertical polymeric nano- or micropillars provide a controlled 3D-environment for measuring cell traction forces^15, 19, 20^, studying cell deformation^21^, tuning cell alignment^14, 22, 23^ or controlling stem cell differentiation^14, 24–26^.

**Figure 1:**
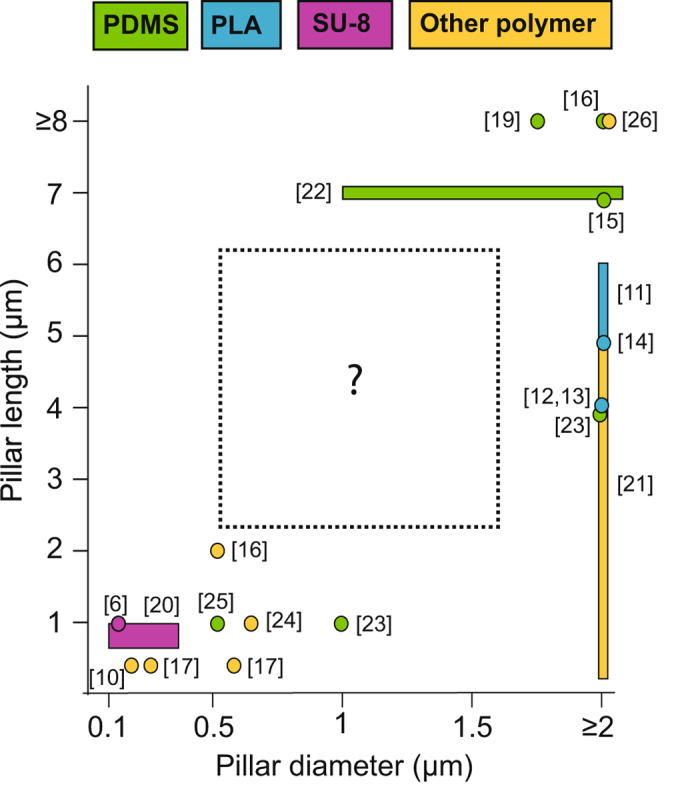
Overview of polymeric pillar geometries used for cell studies. ‘Other polymers’ include PLGA, PUA, PC and PS. PDMS = polydimethylsiloxane, PLA = poly(lactic acid), PLGA = poly(lactic-co-glycolic acid), PUA = poly(urethane acrylate), PC = polycarbonate, PS = polystyrene. See also SI Table S1.

However, a current limitation is the fabrication of vertical arrays of polymeric nanopillars (NPs) on demand for investigation of the influence of NP geometry and distribution on cell behavior. Whereas numerous fabrication approaches have been implemented to generate polymeric micro- and nanopatterns^4^, they usually involve laborious multi-step processing and require expensive masks, especially when submicron features are targeted. Furthermore, most of them are adapted for the nano- or microregime, rarely for both. Indeed, as can be seen from the literature overview of polymeric pillar geometries used for cell studies in Fig. 1 (see SI Table S1 for more details), thinner polymeric pillars (≤500 nm diameter) typically only reach lengths of 1–2 µm, whereas longer structures are seen mainly for diameters in the microregime (≥1 µm). Thus, cell behavior on longer polymeric NPs or for NP diameters in the transition between nano- and microregimes remain only scarcely investigated.

In this context, 3D direct laser writing (3D DLW) by multi-photon polymerization offers an appealing approach to overcome these limitations. It is now well established that this maskless technology enables the production of complex and arbitrary 3D structures both at the micro- and nanoscale^27–30^. In particular, 3D DLW allows for a rapid prototyping of NP arrays with a variety of diameters, lengths, densities and lattice types, which makes it ideal for screening the impact of geometrical parameters on cell behavior.

Here, we take advantage of the great flexibility of 3D DLW to venture into an unexplored size regime, which is difficult to reach with other fabrication techniques. We hypothesize that cells will remain sensitive to geometrical tuning within this regime and strive to extend the Cell Interface with Nanostructure Arrays (CINA) model, which has previously been successfully applied to nanostructures with diameters ≤500 nm^31^. Importantly, if the CINA model applies to this size regime, the ability to predict the cell-NP interface at a given geometry, which can potentially influence the cell response, would make screening and optimization of NP arrays even more rapid. For this purpose, we tune both NP length and density and observe the effects on the interface and behavior of fibroblasts (NIH3T3), which are major players in wound healing^32^ and known to respond to both nano- and microtopographical cues^33, 34^.

## Results and Discussion

### Fabrication of Vertical Polymeric NPs

Polymeric NPs were fabricated by 3D DLW where polymeric 3D microstructures can be precisely defined by displacing the focused laser beam into the photoresist. The submicron resolution is given by taking advantage of the non-linear chemical response of the photoresist combined with the non-linear multi-photon absorption process^35^. Thanks to the exquisite confinement of the photopolymerization reaction, the thickness of the monomer layer does not have to be precisely controlled, which in turn simplifies the experimental procedure (see experimental section). Besides, since the fabrication area in the present paper is limited to a small number of 250 × 250 µm^2^ arrays of NPs, 3D DLW can be seen as a rapid prototyping method to generate various geometrical environments. Indeed, contrary to conventional lithography technologies where a mask is requested, the flexibility of design offered by 3D DLW allows for a rapid screening of different geometries to obtain fast feedback regarding the targeted application^36^.

As a photoresist, we selected a formulation based on pentaerythritol triacrylate (PETIA), which is a multifunctional monomer to favor the elaboration of mechanically stable 3D structures. Besides, Klein and coworkers have demonstrated that PETIA is biocompatible and promotes cell adhesion^18^ making it an ideal candidate for our present investigation. However, contrary to Klein *et al*., we used Lucirin TPO as the photoinitiator, which is more soluble in the chosen monomer and well-adapted regarding the wavelength selected (800 nm) for triggering the photopolymerization^37^.

As mentioned previously, 3D DLW has been exploited for its high flexibility in the design of the NPs both in terms of structure diameter, density, length and pattern, where several parameters may even be altered within a small area on the same chip. Currently, lengths of up to 6 µm and diameters down to ~500 nm (see SI Fig. S1-1) are easily achieved. In the present study, we fix the NP diameter to ~750 nm and explore the effects on NIH3T3 cells when tuning both NP length (3, 6 µm) and density (12, 6, 4, 3, 2 and 1.5 µm center-to-center spacing) inside the unexplored geometry gap marked in Fig. 1. For this purpose, several 250 × 250 µm^2^ arrays of vertical polymeric NPs were made with these specific features. The fabrication time of the different arrays ranges from few tens of seconds to 40 min (see SI Table S2). It has to be noted that despite the serial nature of the direct laser writing process, the fabrication time is reasonable even for dense arrays, which reinforces its character as a rapid prototyping technology.

**Figure 2:**
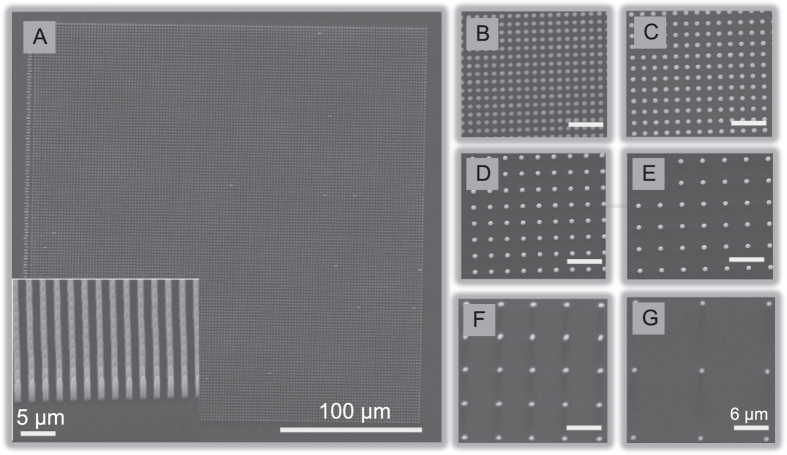
Examples of NP arrays used in the paper. (**A**) Overview of a full 250 × 250 µm^2^ NP array and an inset of a tilted SEM view from the edge of an array. (**B**–**G**) Different polymeric NP center-to-center spacings: 1.5 µm (**B**), 2 µm (**C**), 3 µm (**D**), 4 µm (**E**), 6 µm (**F**), and 12 µm (**G**). Scale bars in B-G all represent 6 µm. All NPs shown here have length 3 µm.

Arrays with the same NP length, but different NP densities, were fabricated within the same sample, so that cell studies could be performed in parallel for several conditions. In Fig. 2, we show scanning electron microscopy (SEM) images of arrays of 3 µm-long NPs at the full range of spacings tested in the cell studies described in the following. Figure 2A exhibits an overview of a 250 × 250 µm^2^ array of NPs achieved by TPS. The inset corresponds to an enlarged and tilted view of the same array. Figure 2B–G represent an enlarged view of several arrays with specific center-to-center spacing of 1.5, 2, 3, 4, 6 and 12 µm respectively. In SI section S1, SEM images of both NP lengths at different densities are shown (SI Fig. S1-2) along with statistical length measurements performed for both sample types (SI Table S3).

### Cell adhesion on NPs

**Figure 3:**
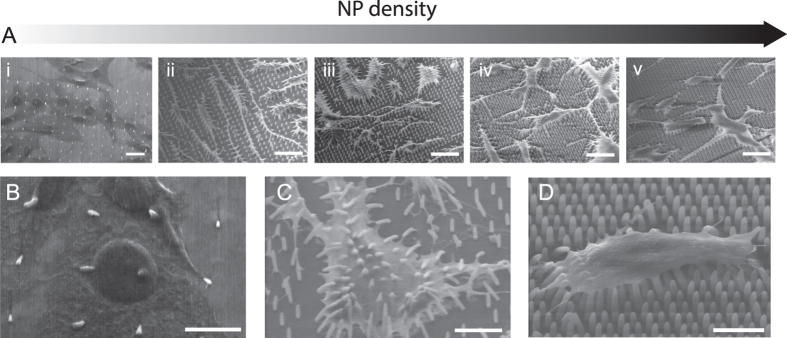
Overview of cell morphology on NPs of increasing density. (**A**) SEM overview images of NIH3T3 cells on 12 (i), 4 (ii) 3 (iii), 2 (iv) or 1.5 (v) µm NP spacing. NP lengths are l~3 µm for 12 µm spacing and l~6 µm for spacings 4–1.5 µm. Scale bars represent 20 µm. (**B**–**D**) Zoom-ins on single cells on 12 (**B**), 4 (**C**) or 1.5 (**D**) µm NP spacing. Scale bars represent 10 µm (**B**,**C**) or 5 µm (**D**).

Fibroblasts (NIH3T3) were grown on the NP arrays created by 3D DLW and cell adhesion on this new range of geometries was first evaluated by SEM. The SEM images in Fig. 3A provide an overview of NIH3T3 cell morphology on NPs of increasing density. Starting at 12 µm spacing (i), the cell morphology seems unaltered when comparing to the cells on the NP-free area in the upper part of the image, but going across 4 µm (ii), 3 µm (iii), 2 µm (iv) and finally 1.5 µm (v) spacing, the cell morphology is clearly changing (see SI Figure S2 for a quantitative analysis).

When inspecting zoom-ins of single cells on spacings 12 µm (B), 4 µm (C) and 1.5 µm (D), the type of interface also seems to gradually change from the cells having a large contact area with the flat substrate between NPs on the lowest density and to adhesion only at the upper parts of the NPs on the highest density. The cell interface thus follows the trend observed also on thinner polymeric^6^ or silicon^38^ nanopillars as well as on indium arsenide^39^ or gallium phosphide^40^ nanowires.

The cell-NP interface was furthermore visualized through fluorescent staining of the membrane and cytosol in live NIH3T3 cells (SI Figure S3), which confirmed that a tight interface is formed through plasma membrane deformation around each NP under the cell.

### Cytoskeletal Remodeling

**Figure 4:**
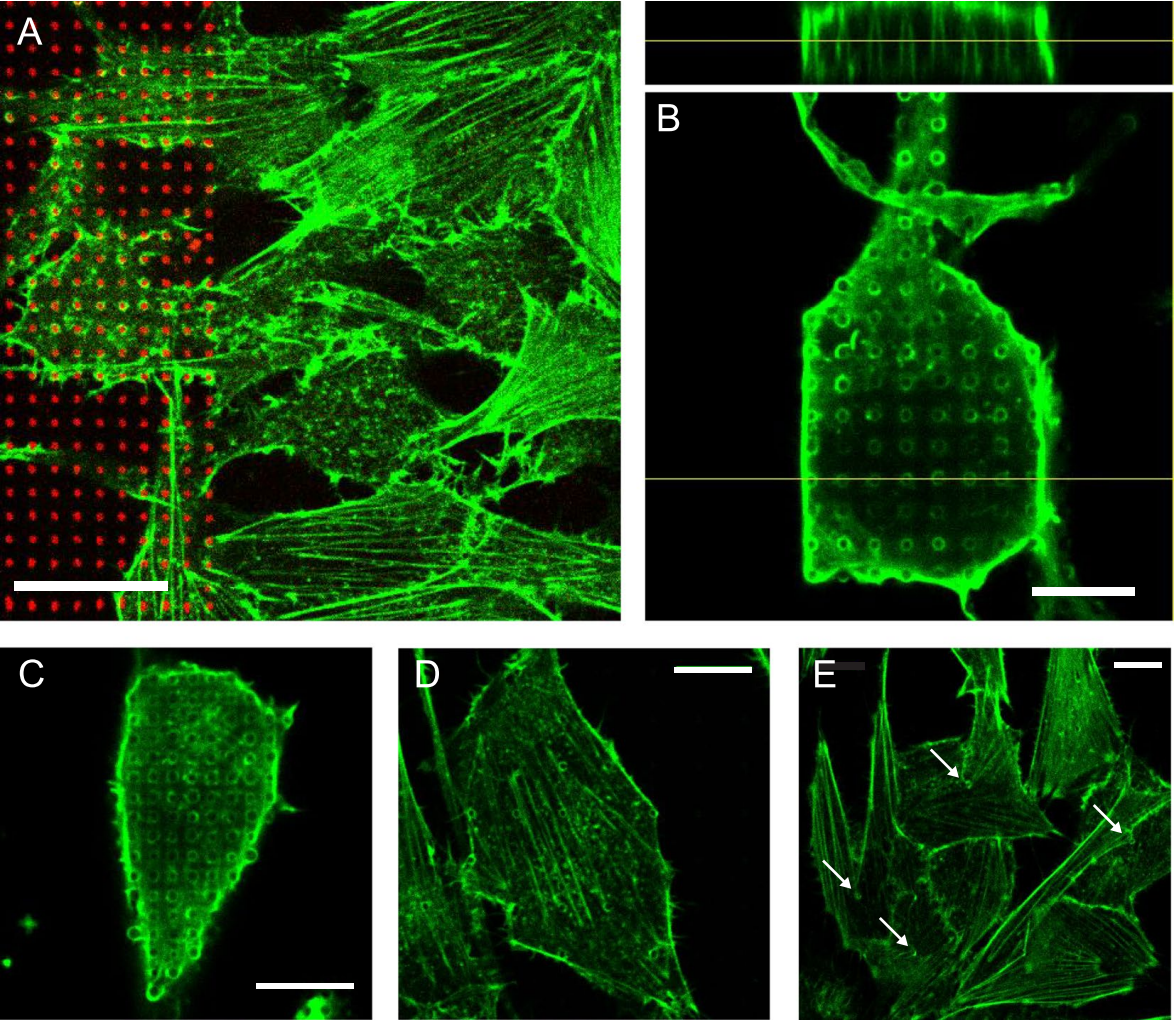
Actin remodeling on NPs. (**A**) NIH3T3 cell actin structure (green) at the border of the NP array (NPs in red) with 3 µm spacing. The scale bar represents 20 µm. (**B**) Confocal slice through the actin signal of a single cell on 3 µm-spaced NPs (l ~ 6 µm) and an orthogonal side view through the confocal stack in the indicated position. The scale bar represents 10 µm. (**C**–**E**) Confocal slices through the actin signal of single cells on 2 (**C**), 6 (**D**) or 12 (**E**) µm NP spacings. Positions of clear NP-actin colocalization are indicated with arrows in (**E)**. Scale bars represent 10 µm.

To reveal the effect of the tight interface on the cytoskeleton, the actin filaments were visualized through immunostaining. In Fig. 4A, the transition from the NP-free area and onto 3 µm-spaced NPs is shown and it is clear that the normal cytoskeletal organization with long parallel fibers is disturbed. From the closer look at a single cell on NPs in Fig. 4B, the cause of the disturbance is clear, as the actin filaments are seen to tightly wrap each NP both under the cell and along the cell edges. This actin-NP colocalization is even observed on very high densities such as 2 µm spacing (C). However, the effect is less prominent on lower densities such as 6 µm (D) and 12 µm (E) spacing, where the overall cytoskeletal structure appears preserved although a few cases of colocalization with NPs are seen. This is in good agreement with observations for stem cells on a PDMS NP density gradient, where the cytoskeletal arrangement was found to only be altered below 5.6 µm spacing^24^. In addition to being dependent on the NP spacing, the remodeling of the cytoskeletal structure in favor of colocalization with NPs seems to also increase with NP length (see SI Figure S4). The kind of actin filament colocalization observed here has also been documented with HeLa cells on SU-8 NPs^6^ and may therefore not be specific to the cell type and material used in the present paper.

### Cell Settling Height

From the SEM images in Fig. 3, it is evident that cells settle very differently and deform to different extents around the NP depending on the spacing of these. The difference in cell perturbation and immediate nanotopography seen by each cell could thus potentially influence cell behavior. Furthermore, different types of cellular applications require different types of interfaces, so understanding and predicting cell settling as a function of nanostructure geometry is highly desirable for appropriate design of nanostructure arrays. To this end, we have previously developed a theoretical tool, the Cell Interface with Nanostructure Arrays (CINA) model, which predicts the cell settling height as a function of nanostructure density, length and diameter^31, 39^. So far, the CINA model has been successfully applied for nanostructure diameters of ≤500 nm and here we extend the model to the present structures with diameters of ~750 nm (see SI section S5).

**Figure 5:**
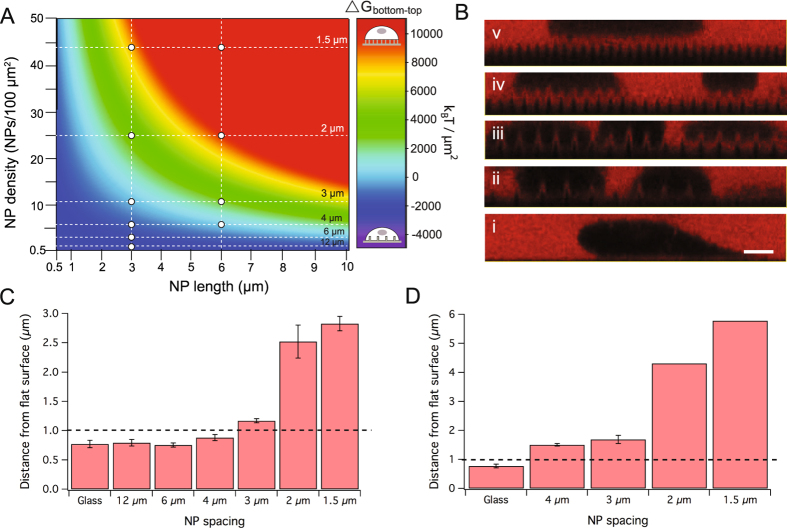
Theoretical and experimental cell settling height on NPs. (**A**) The free energy difference, ΔG_bottom-top_, between ‘bottom’ and ‘top’ state cell settling (see sketches) calculated as a function of NP length and density using the CINA model for a fixed NP diameter of 750 nm. The combinations of NP length and density explored in the present paper are marked. (**B**) Representative confocal side views of NIH3T3 cells imaged in a solution of cell-impermeable dye on glass (i) or 3 µm-long NPs spaced by 4 (ii), 3 (iii), 2 (iv) or 1.5 µm (v). The scale bar (i) represents 5 µm. (**C**,**D**) Average cell settling heights measured from side views as those in B for NP lengths 3 µm (**C**) or 6 µm (**D**) and different NP densities. A NP-free area (glass) was used as a reference. The error bars represent the standard error of the mean between at least two independent experiments (data for spacings 2 and 1.5 µm in (**D**) stem from only a single experiment, but the standard deviation between settling heights for single cells was <1 µm in each case). A cell settling height of 1 µm (marked with a punctured line) is defined as the border between ‘top’ and ‘bottom’ settling states for comparison with the predictions of the CINA model.

Figure 5A shows the free energy difference, ΔG_bottom-top_, between a cell deforming and contacting the flat substrate between NPs (‘bottom’) and a cell settling at the very tips of the NPs (‘top’) as a function of NP length and density (fixed diameter = 750 nm). For negative values of ΔG_bottom-top_, ‘bottom’ settling is energetically favorable and for positive values of ΔG_bottom-top_, ‘top’ settling is favorable. Along the light blue line marking ΔG_bottom-top_ = 0, the two states are equally probable and the corresponding density is the so-called ‘crossover density’ for the given combination of NP length and diameter. The combinations of density and length investigated in the present paper have been marked and it is seen that for a NP length of ~3 µm, the spacings 12–4 µm are below the crossover density, and spacings 2–1.5 µm are above, while a spacing of 3 µm corresponds exactly to the predicted crossover density (11 NPs/100 µm^2^). For a NP length of ~6 µm, a spacing of 4 µm corresponds to the predicted crossover density (6 NPs/100 µm^2^), while spacings 3–1.5 µm are all above.

To verify these predictions, the extracellular space was stained with a cell-impermeable dye to reveal the distance between the cells and the flat substrate between NPs. Representative confocal side views of cells are shown in Fig. 5B for glass (i) and for 3 µm-long NPs with increasing density (4 µm to 1.5 µm spacing). The observed transition from a pure ‘bottom’ state (ii), over mixed settling (iii) to a pure ‘top’ state (iv, v) is in good accordance with the CINA model prediction. Figure 5C and D show the average cell settling heights measured for the different NP spacings with NP lengths 3 µm or 6 µm, respectively. We have previously defined a cell settling height of 1 µm to mark the border between ‘bottom’ and ‘top’ settling (including partly deformed states) and the transition is seen to happen between 4 and 3 µm spacing for 3 µm length, whereas it is already happening at 4 µm spacing with 6 µm-long NPs, both as predicted by the CINA model. A generic predictive tool for the extended CINA model is found in SI Figure S6.

### Guiding of cell growth using NP arrays

**Figure 6:**
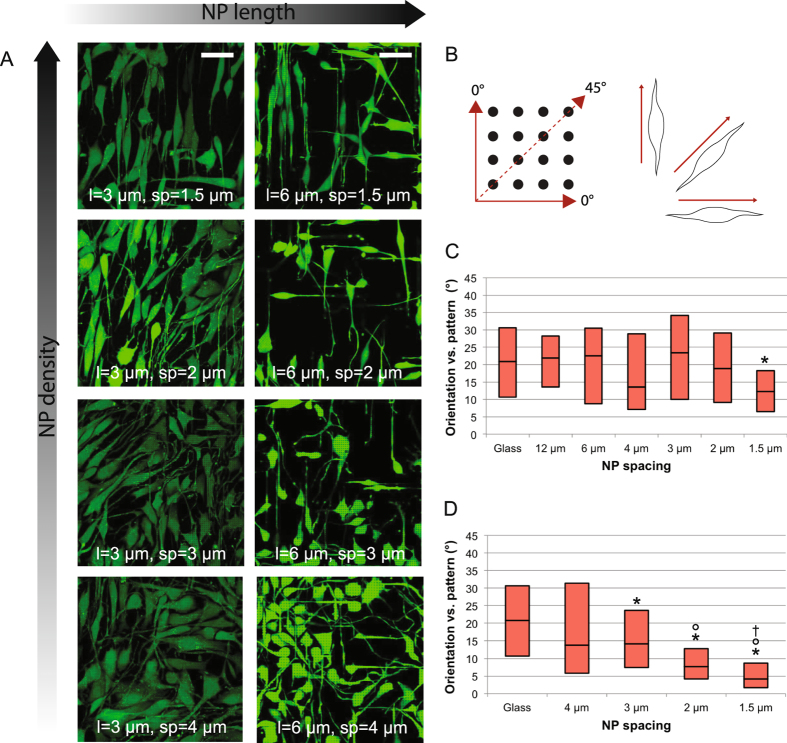
Cell alignment on NPs. (**A**) Overview of NIH3T3 cells seen by a cytosolic stain (green) on NPs with the indicated lengths and spacings. The scale bar in the upper left image represents 50 µm and applies to all. (**B**) Cells from at least two independent experiments (typically ~60 cells in total) were analyzed according to the orientation between their major axis and the NP pattern as defined in the sketch, which is oriented as in the overviews of fluorescently stained cells. (**C**,**D**) Boxplots showing the distribution of cell orientations according to the NP pattern as defined in B for different NP spacings and NP lengths 3 µm (**C**) or 6 µm (**D**). A NP-free area (glass) was used as a random orientation control. The data for each condition stems from at least two independent experiments with orientations quantified for ~60 cells in total. Box = 25^th^ and 75^th^ percentiles; line = median. The boxplot whiskers are not shown, since they all go down to 0° (minimum value in distribution) and up to 45° (maximum value in distribution). The corresponding histograms are found in SI Fig. S7-1 and S7-2. Significant differences (p < 0.05) are indicated as * (vs. glass), ° (vs. 4 µm spacing), and † (vs. 3 µm spacing).

The tight interface between NIH3T3 cells and NPs observed with SEM and fluorescence imaging of the cell membrane and actin filaments could be beneficial for guiding of cell growth. To evaluate the cell alignment potential of the polymeric NPs, fluorescence overview images of NIH3T3 cells on NPs (Fig. 6A) were analyzed to extract the orientation of the major axis of single cells with respect to the NP pattern (Fig. 6B).

The distributions of cell orientations have been plotted for NP lengths 3 µm and 6 µm, respectively, in Fig. 6B and C. For the NP-free control (glass), the median of the distribution is close to 22.5° as expected for a random orientation. For the shorter NPs (Fig. 6C), the distributions remain broad and quite similar to glass until the highest density (1.5 µm spacing), where a narrowing and clear shift towards alignment with the pattern (0°) is seen. For the longer NPs (Fig. 6D), this shift happens gradually starting from 4 µm spacing and alignment is already efficient on 2 µm spacing. The corresponding histograms are found in SI section S7. It is noteworthy that alignment on the diagonal (45°) is not observed to the same extent, which suggests that the cell filopodia preferably follow the direction of shorter NP spacing as also observed for neurites on an anisotropic micropillar array^41^.

Increasingly efficient cell alignment has also been demonstrated on micron-wide polymeric lines with decreasing spacing for both endothelial^7^ and NIH3T3^34^ cells. However, in addition to NP spacing, the NP length is also found to have a profound effect on cell alignment efficiency. In fact, cell alignment efficiency appears to be correlated with cell settling height, when the tendencies of the data in Figs 5 and 6 are compared. Thus, cell settling states where cells are only in contact with the NPs, and preferably only the very tips of these, seem to promote cell alignment. Such clear guiding at the tips was also observed with SEM, where even outgrowths of cells mainly adhering on the flat substrate next to the NP area were seen to climb and be guided at the tips of the NPs (SI Figure S8). This is in good agreement with the thorough study by Bucaro *et al*. of cell alignment versus pillar array geometry, where cell alignment was shown to be very efficient for cells adhering only at the tips of both silicon and polymeric pillars with spacings in the range 2 µm-1.5 µm^38^. However, they found that the effect wears off again towards even higher pillar densities, so there might be an upper limit to the alignment-promoting effect of a high NP density.

Since cell alignment efficiency can thus be linked directly to the cell settling height, it is possible to design an optimized NP array for the desired tuning of cell alignment through theoretical predictions of the cell settling. We therefore envision that the combination of the CINA model with the flexibility of the 3D DLW demonstrated in the present paper holds the potential of immensely improving the use of polymeric structures for cell guiding.

## Conclusion

Here, we have demonstrated that 3D DLW can be used for fast and flexible prototyping of polymeric NPs from PETIA, which allowed us to systematically study the effect of NP array geometry (density and length) on the behavior of NIH3T3 cells. We have shown that the polymeric NPs generally provide a biocompatible environment for cell adhesion and sustained cell growth, and that the cells form a tight interface with the NPs through both direct membrane adhesion and actin remodeling around the NPs. As a consequence of this tight interaction, the cell morphology, settling height and alignment with the NPs can be tuned by changing the NP array geometry. In fact, the cell settling height, which can be predicted by an extended version of our previously published CINA model, appears to correlate with the cell alignment efficiency on a given NP array, with cells adhering only to the tips of the NPs being more efficiently aligned with the NP pattern. Thus, in addition to an increased understanding of cell behavior as a function of NP array geometry, the combination of the CINA model with the flexibility of the 3D DLW form a potent tool for designing future polymeric NP arrays for improved cell guiding.

### Experimental Section

#### Fabrication of vertical NPs by 3D DLW

NPs were fabricated using a femtosecond laser source (Chameleon Ultra II, 140 fs @ 800 nm, from Coherent) combined with a TeemPhotonics microfabrication 3D system as described previously^42^. The formulation contained 3% w/w of photoinitiator (Lucirin-TPO from BASF, Ludwigshafen, Germany) in monomer (pentaerythritol triacrylate (PETIA), from Sigma-Aldrich) and a few hundred microliters were extracted with a Pasteur pipette and dropped on a glass coverslip. The laser beam was focused into the photoresist via an objective lens (40x, NA: 0.65) and typical fabrication was performed with an optimized exposure time and laser power of 10 ms and 10 mW, respectively (see SI Fig. S1-1). After laser exposure, the sample was immersed in ethanol (analytical grade, Sigma-Aldrich) for 15 min to remove the non-polymerized material. Finally, samples were dried with nitrogen and stored in glass vials until use.

#### Cell culture

Flp-in 3T3 cells (Invitrogen) with a gene coding for a membrane-anchored SNAP-tag at the Flp-in site (will be referred to as NIH3T3) were maintained at 37 °C, 5% CO_2_, and >95% humidity in DMEM/F-12 Glutamax-I medium (Gibco) supplemented with 10% calf serum (Sigma) and 100 µg/ml hygromycin B (Invitrogen).

#### Interfacing of cells with NPs

Prior to cell interface, the NP array was washed for 2 × 30 min. in excess MQ H_2_O to remove any residual chemicals, sterilized with 70% EtOH and washed 3x with cell medium. The NIH3T3 cells were added dropwise to the NP array at a density of 60,000 cells/cm^2^ and grown in medium supplemented as above for 24 h prior to analysis.

#### SEM imaging

The NIH3T3 cells were fixed with 4% paraformaldehyde (PFA, Sigma) and dehydrated with methanol as previously described^43^. The samples were then sputter-coated with 5 nm Au and SEM images were collected using a JEOL JSM-6320F with 10 kV acceleration voltage. Cell-free imaging of the NPs was performed directly using a FEI-Quanta 400 model with 15 kV acceleration voltage.

#### Immunostaining of actin

The NIH3T3 cells were fixed with 4% PFA for 1 h at room temperature (RT) and then permeabilized and blocked with 0.25% Triton X-100 (BioChemika) and 1% bovine serum albumin (Sigma) for 20 min. prior to incubation with 2% rhodamine-phalloidin (Molecular Probes) for 40 min. at RT.

#### Fluorescence imaging

Both live and immunostained NIH3T3 cells were imaged with an inverted confocal microscope (Leica TCS SP5) using a 63x magnification, water-immersion objective with numerical aperture 1.2. For 12–3 µm spacing, the sample could be imaged from below, but it was inverted for imaging on 2–1.5 µm spacing.

#### Cell settling height study

NIH3T3 cells were imaged in 100 µM ATTO647 (ATTO Technology, Inc., New York, USA) with an inverted confocal microscope (see above). The distance between the apical side of the cell and the flat substrate between NPs was measured using vertical cross sections through z-stacks of the confocal images (“side views”) in ImageJ software. Typically, >10 cells were analyzed per condition and replicate.

#### CINA model calculations

The free energy difference between the ‘bottom’ and ‘top’ cell settling state was calculated from ref. 31:

$${\rm{\Delta }}{G}_{bottom \mbox{-} top}=-w\cdot {\rm{\Delta }}{A}_{c,bottom \mbox{-} top}+\sigma \cdot {\rm{\Delta }}{O}_{bottom \mbox{-} top}+{\rm{\Delta }}{G}_{b,bottom \mbox{-} top}$$

where Δ*A*_*c,bottom-top*_ is the extra surface contact area gained from deforming the membrane along the NPs and contacting the flat substrate, and Δ*O*_*bottom-top*_ and Δ*G*_*b,bottom-top*_ are the extra exposed surface area and bending energy, respectively, associated with the deformation around the NPs. *w* is the specific adhesion energy per unit area, which was adjusted to 1.2·10^−17^ J/µm^2^ (see SI section S5), and σ is the surface tension, which was fixed to 2.4·10^−17^ J/µm^2^ as previously. The bending modulus, κ, which enters into the Δ*G*_*b,bottom-top*_ term, was fixed to 9·10^−19^ J as previously^31^.

#### Cell alignment analysis

The cytosol of live NIH3T3 cells was stained with 3 μM calcein acetoxymethyl (Molecular Probes). CellProfiler software (version 2.0, Massachusetts Institute of Technology, Cambridge, MA) was used to identify each cell from the cytosolic live-cell signal (see above) and then calculate the orientation of the major axis of each cell with a horizontal orientation as the reference. Data stem from at least two independent experiments and ~60 cells were typically analyzed per condition. Cell orientation distributions for different NP array geometries were compared using Student’s unpaired t test with a two-tailed distribution, where a p-value below 0.05 was considered a significant difference.

### Data availability

The datasets generated during the current study are available from the corresponding author on reasonable request.

## Electronic supplementary material

Supporting Information
