# Supplementary material for: Rapid Prototyping of Polymeric Nanopillars by 3D Direct Laser Writing for Controlling Cell Behavior
Source: Sci Rep. 2017 Aug 23;7:9247. doi: 10.1038/s41598-017-09208-y (PMC5569057; doi:10.1038/s41598-017-09208-y)
Supplement: Supplementary file 1 — Supporting Information [file 41598_2017_9208_MOESM1_ESM.pdf]

Supporting Information

Rapid Prototyping of Polymeric Nanopillars by 3D Direct Laser Writing for Controlling Cell Behavior

Nina Buch-Månson, Arnaud Spangenberg, Laura Piedad Chia Gomez, Jean-Pierre Malval, Olivier Soppera, Karen L. Martinez**

Table S1. Overview of polymeric pillars used in cell studies, including pillar material, geometry and cell types. PDMS=polydimethylsiloxane, PLA=polylactic acid, PLGA=poly(lactic-co-glycolic acid), PUA=poly(urethane acrylate), PC=polycarbonate, PS=polystyrene.

Material	Diameter	Length	Spacing	Cell type	Reference
PDMS	500 nm	1 µm	3-10 µm	Stem cells	[1]
	2 µm	6.9 µm	2 µm	NIH3T3	[2]
	1, 4 µm	1, 4 µm	4, 1 µm	Primary fibroblasts	[3]
	1.8 µm	8.3 µm	4 µm	NIH3T3	[4]
	1-3 µm	7 µm	2 µm	BAEL	[5]
	5 µm	20 µm	4-12 µm	Ref52 fibroblasts	[6]
PLA	2-15 µm	5-6 µm	2-20 µm	Cancer cells	[7]
	3 µm	5 µm	>10 µm	Stem cells	[8]
	7 µm	4 µm	7 µm	Cancer cells	[9,10]
PLGA	3 µm	0.2-5 µm	6 µm	Stem cells	[11]
PUA	700 nm	1 µm	1.2-5.6 µm	Stem cells	[12]
	300, 600 nm	N/A (~0.5 µm)	0.3-4.2 µm	Cancer cells	[13]
PC	500 nm, 2 µm	2, 10 µm	0.5, 2 µm	Stem cells	[14]
PS	200 nm	0.5 µm	0.5 µm	Stem cells	[15]
SU-8	100-380 nm	0.6-1 µm	400, 880 nm	CHO	[16]
	120 nm	1 µm	0.75-5 µm	HeLa	[17]

S1. Polymeric NP Samples

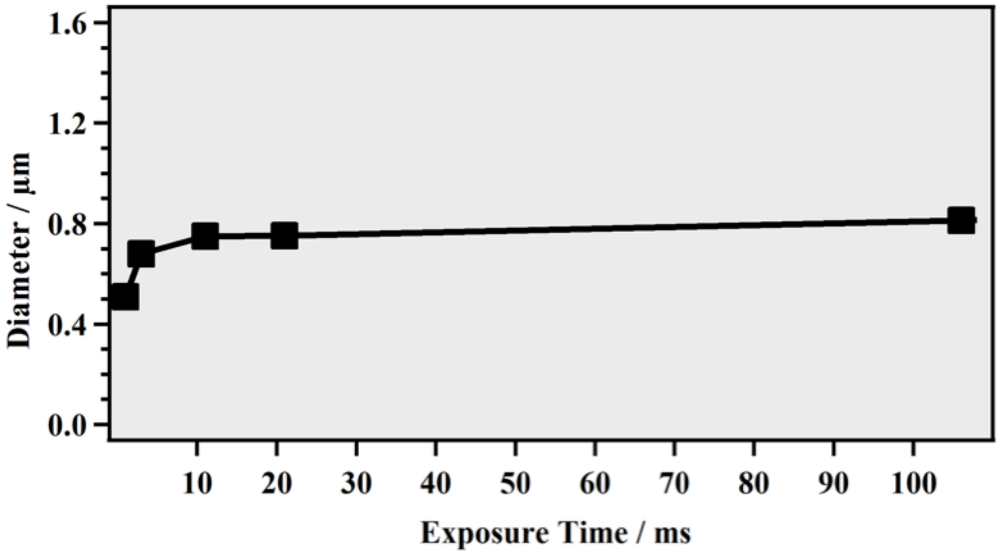

Figure S1-1. Impact of exposure time on NP diameter at a fixed power. The NP diameter can be tuned from 500 nm to 750 nm by adjusting the exposure time from 1 ms to 110 ms. Prototyping of 250 x 250 µm^2 arrays of NPs has been performed with laser power fixed arbitrarily to 10 mW. Exposure time has been set to 10 ms in order to keep a reasonable fabrication time and to investigate a diameter (~750 nm) corresponding to a rarely explored size regime.

Table S2. Fabrication time for each 252 x 252 µm^2 array of NPs with an exposure time set to 10 ms.

		**Density (center-to-center spacing)**					
		1.5 µm	2 µm	3 µm	4 µm	6 µm	12 µm
**Number of NPs**		28224	15876	7056	3969	1764	441
**Length**	3 µm	20 min	11 min 15s	5 min	2 min 17s	1 min 15s	20s
	6 µm	40 min	22 min 30s	10 min	5 min 35s	2 min 30s	40s

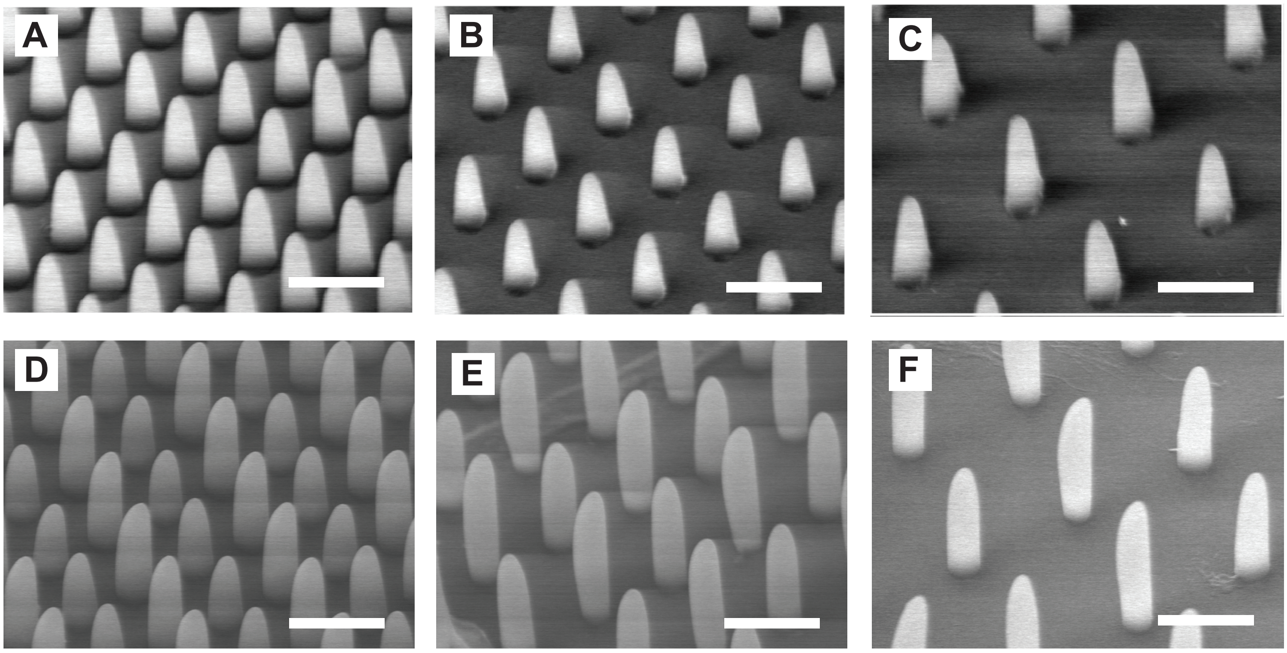

Figure S1-2. SEM images of polymeric NPs of 3 μm (A-C) or 6 μm (D-F) length and spacings 1.5 μm (A,D), 2 μm (B,E) or 3 μm (C,F). The images were collected with a horizontal sample holder in a JEOL JSM-6320F using 10 kV acceleration voltage and a 35° (A-C) or 25° (D-F) tilt. Scale bars represent 2 μm.

Table S3. Statistical length measurements performed on SEM images like in Figure S1 for 3 μm spacing samples with NP target lengths 3 or 6 μm.

NP target length	Measured average	Standard deviation	No. of NPs measured
3 μm	3.01 μm	0.28 μm	62
6 μm	5.95 μm	0.53 μm	48

S2. Cell spreading on different NP array geometries

Cells were stained with calcein-acetoxymethyl to reveal their outlines and imaged in a confocal fluorescence microscope (same data set as used for cell orientation analysis in main text Figure 6). Cell Profiler software was used to extract the spreading area and elongation (major/minor axis) of single cells and the results are summarized in **Figure S2**. While the cell spreading area is not trivially correlated with the NP spacing, a clear trend for increasing cell elongation with decreasing spacing is seen for both NP lengths.

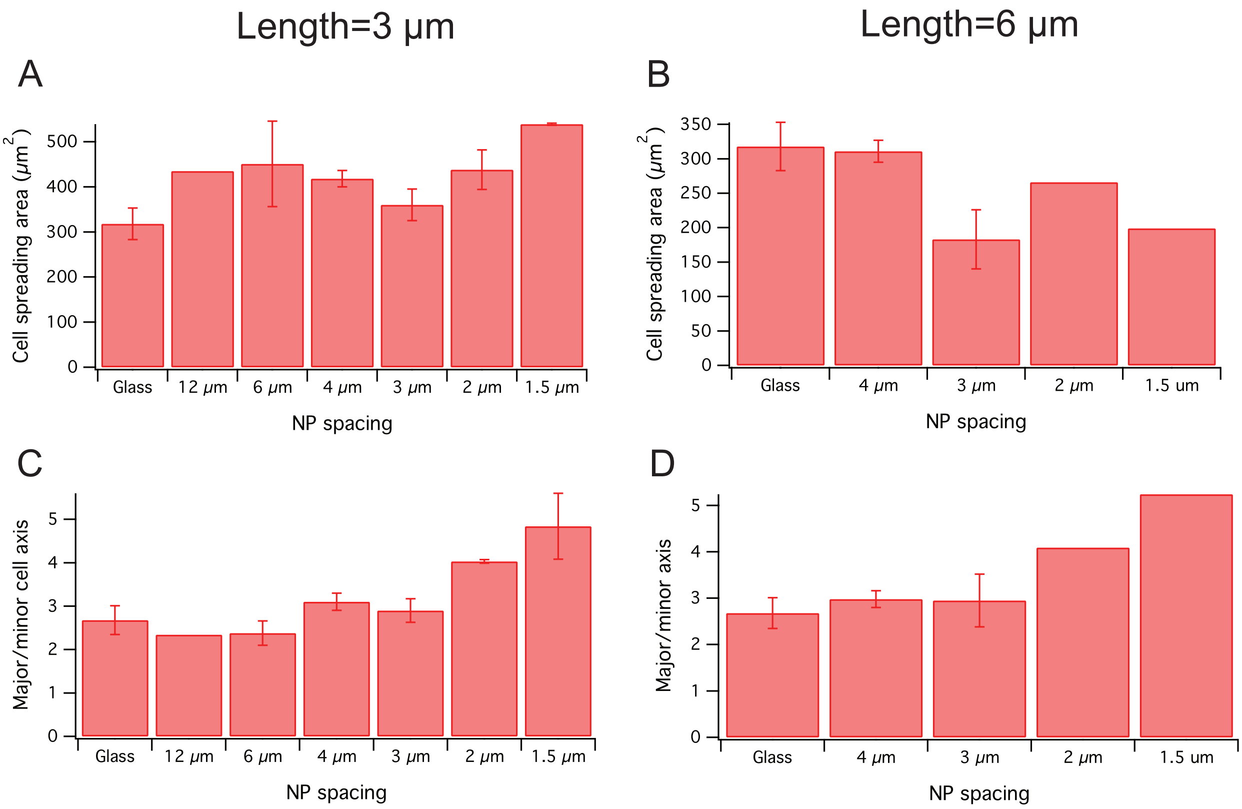

Figure S2. Cell spreading on different NP array geometries. A,B) Cell spreading area on glass and different spacings of NPs with length 3 or 6 μm as indicated. C,D) Cell elongation (major/minor axis) on glass and different spacings of NPs with length 3 or 6 μm as indicated. The error bars represent the standard error of the mean between at least two independent experiments, while no error bars indicate that only a single experiment (with multiple cells analyzed) has been performed.

S3. Plasma membrane conformation at cell-NP interface

To visualize the cell interface at the NPs in more detail, we stained the membrane of live NIH3T3 cells via a stably expressed extracellular SNAP-tag with 5 µM SNAP-Surface 549 as previously described.[18] Labeling with the cell-impermeable dye ensured that only the extracellularly exposed plasma membrane was fluorescently stained. The cells were furthermore stained with a cytosolic live-cell indicator (calcein acetoxymethyl).

Figure S3A shows a confocal slice trough the membrane signal of a cell on 4 µm spacing, where the membrane is clearly seen to tightly deform around each NP under the cell. Above are orthogonal side views through both the membrane (yellow) and cytosolic (green) signals of the confocal stack at the position indicated in the slice below. The NPs can be distinguished through their shadows in the cytosolic signal and it is evident that the cell membrane is deformed along the entire length of the NPs. Such a tight membrane deformation is even observed on very high densities (B,C) and confirms the chemical compatibility between the material and the cell membrane, which allows for direct adhesion on the polymeric NPs.

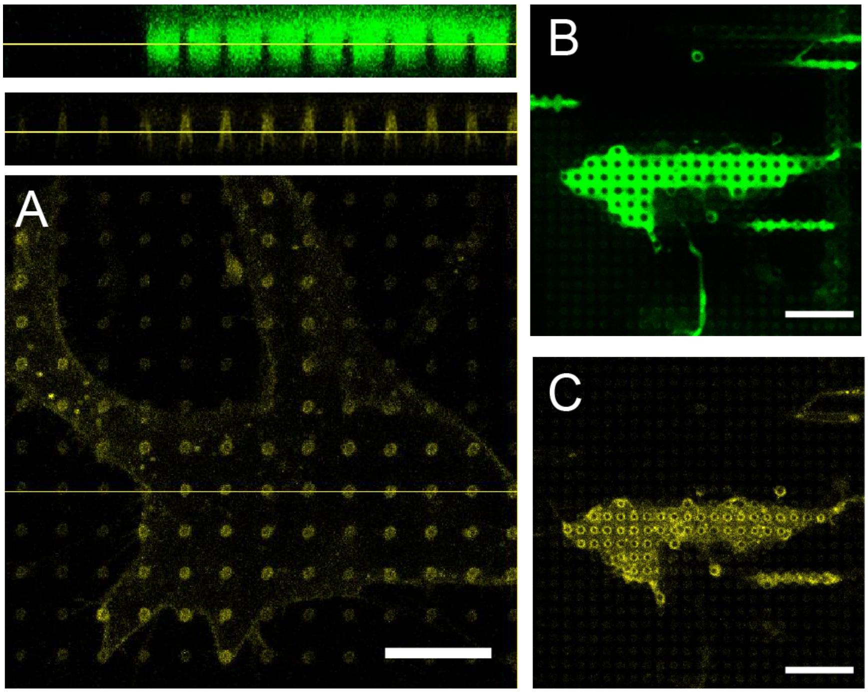

Figure S3. Membrane conformation on NPs. A) Confocal slice through the membrane signal of an NIH3T3 cell on 4 µm NP spacing (l~3 µm) and orthogonal side views through the confocal stack in the indicated position for both the membrane (yellow) and cytosolic live-cell indicator (green) signals. NPs are seen by their shadow in the cell cytosol. B, C) Confocal slices through the cytosolic (B) and membrane (C) signals of a cell on 2 µm NP spacing (l~6 µm). All scale bars represent 10 µm.

S4. Cytoskeleton Remodeling Overviews

Figure S4 shows confocal overview images of NIH3T3 cell actin stained by rhodamine-phalloidin 24 h after interface with polymer NPs with lengths 3 µm (A,C,E,G) or 6 µm (B,D,F,H) and spacings 4 (A,B), 3 (C,D), 2 (E,F) or 1.5 µm (G,H). The colocalization of actin filaments with polymer NPs appears more prominent on longer NPs when comparing with shorter NPs, in particular on 4 and 3 µm spacings.

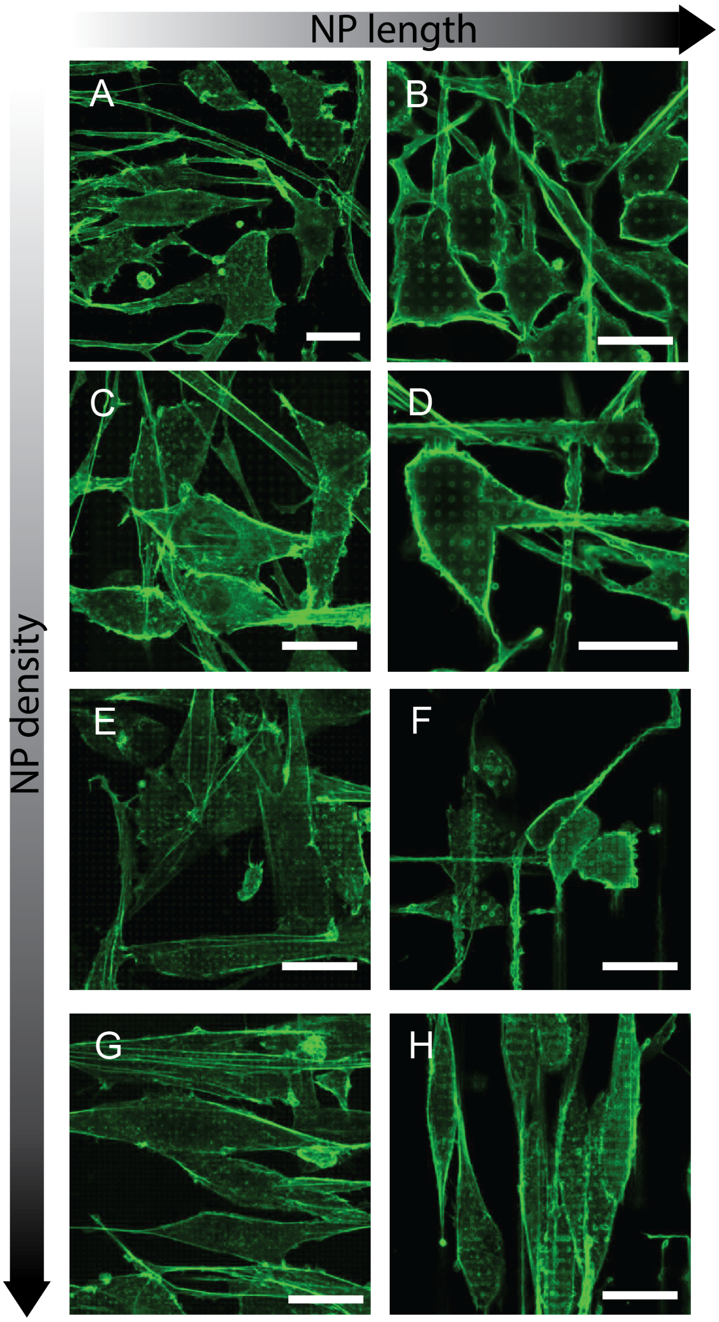

Figure S4. Confocal overview images of NIH3T3 actin structure on NPs with lengths 3 µm (A,C,E,G) or 6 µm (B,D,F,H) and spacings 4 (A,B), 3 (C,D), 2 (E,F) or 1.5 µm (G,H). The images were prepared by summing 3.5 µm of the confocal stacks. Scale bars represent 20 µm.

S5. Adjusting the specific adhesion energy (w) in the CINA Model

In our previously published work, where we explored the predictions of the CINA model for nanostructures with diameters ≤500 nm[19], we fixed the specific adhesion energy (w) to $2.2 \cdot 10^{-17}$ J/µm^2 for most simulations. However, this parameter could realistically change within a range of $0.5\text{-}8 \cdot 10^{-17}$ J/µm^2 depending on the cell type, structure material and coating, which is also explored in the original paper. In the present study, we have tuned the value of w to $1.2 \cdot 10^{-17}$ J/µm^2 since the cell adhesion is typically reduced for untreated polymeric structures[20,21]. This minor adjustment allows for an excellent fit of the observed cell settling on the 750 nm-diameter polymeric NPs as can be seen from Figure 5 in the main text. **Figure S5** shows the free energy difference, $\Delta G_{bottom\text{-}top}$, between 'bottom' and 'top' cell settling as calculated by the CINA model with the original (A) and new (B) value of w. According to the unadjusted plot in Figure S4A, cells should be deformed into the 'bottom' state on all the investigated combinations of spacing and length, except for 6 µm-long NPs spaced by 1.5 µm. According to the adjusted plot in Figure S4B, both 'bottom' state, mixed and 'top' state settling should be observed for the explored points, as is also found to be the case experimentally.

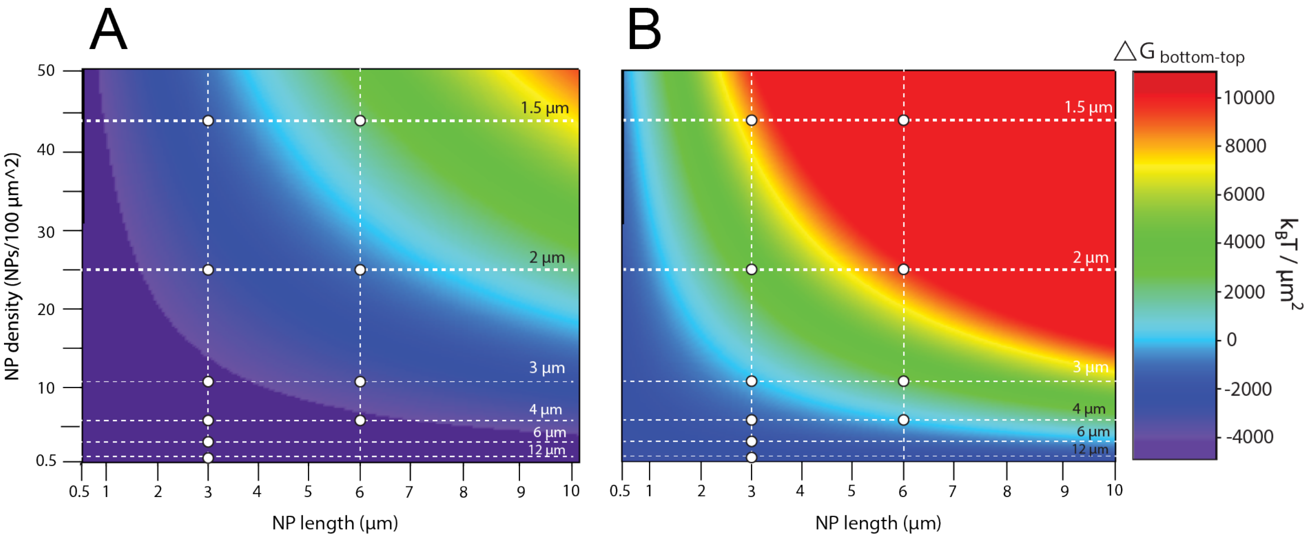

Figure S5. The free energy difference, $\Delta G_{bottom\text{-}top}$, between 'bottom' and 'top' cell settling calculated as a function of NP length and density using the CINA model for specific adhesion energy (w) input values of $2.2 \cdot 10^{-17}$ J/µm^2 (A) and $1.2 \cdot 10^{-17}$ J/µm^2 (B). The combinations of NP length and density explored in the present paper are marked. B is identical to Figure 5A in the main text.

S6. Generic Prediction Tool for the Extended CINA Model

The crossover density of the CINA model[19] marks the border between the density regimes where 'bottom' or 'top' cell settling is more probable and, using the model, it can be estimated for a given combination of pillar diameter and length. In **Figure S6**, an extended CINA model crossover prediction tool is provided for both the original (A) and adjusted (B) values of the specific adhesion energy w (see section S5). The adjusted prediction (B) is anticipated to be more appropriate for polymeric structures.

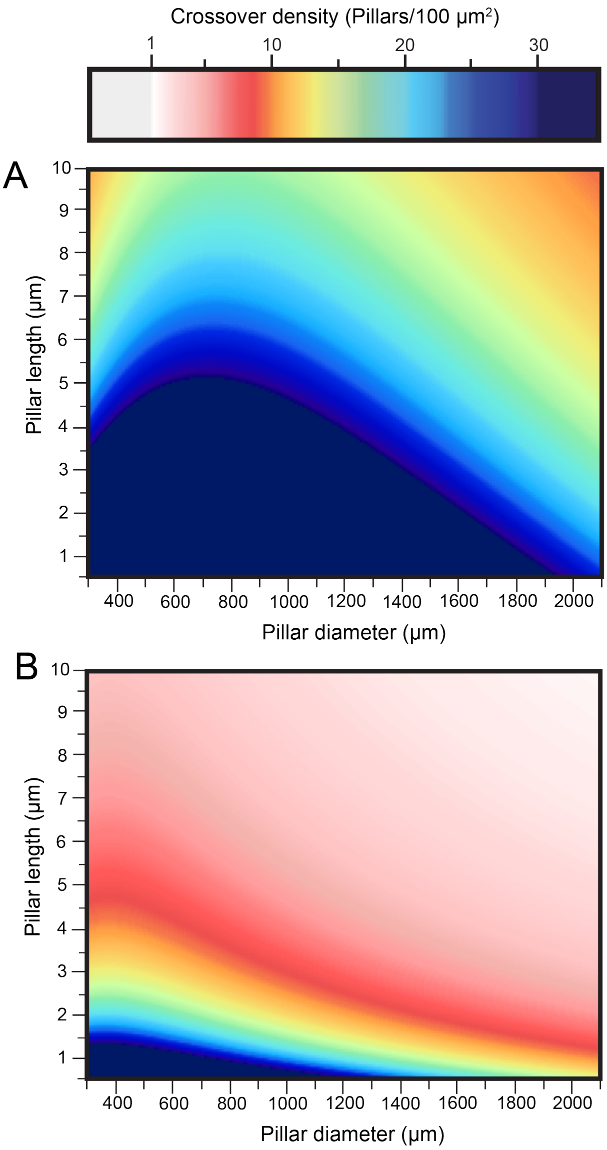

Figure S6. The crossover density predicted by the CINA model as a function of pillar diameter and length. Below the crossover density, $\Delta G_{bottom\text{-}top}<0$ and cell deformation into the pillar array is favorable. Above the crossover density, $\Delta G_{bottom\text{-}top}>0$ meaning that cell deformation is unfavorable and the cells are predicted to stay on top of the pillars. The specific adhesion energy w was fixed to the original value of $2.2\cdot10^{-17}$ J/μm^2 (A) or $1.2\cdot10^{-17}$ J/μm^2 (B) as successfully used for the polymeric NPs in the present paper.

S7. Cell Alignment Histograms

Figure S7-1 shows representative confocal overview images of NIH3T3 cells stained with calcein-AM 24 h after interface on glass (A) or 3 µm-long NPs spaced by 12 µm (C) or 6 µm (F). Histograms of cell orientation according to the definition in A are shown in B, D and F, respectively. **Figure S7-2** shows the histograms of cell orientation for all the conditions shown in Figure 7A. All the histogram data sets shown here are summarized in the boxplots of Figure 6C and D in the main text.

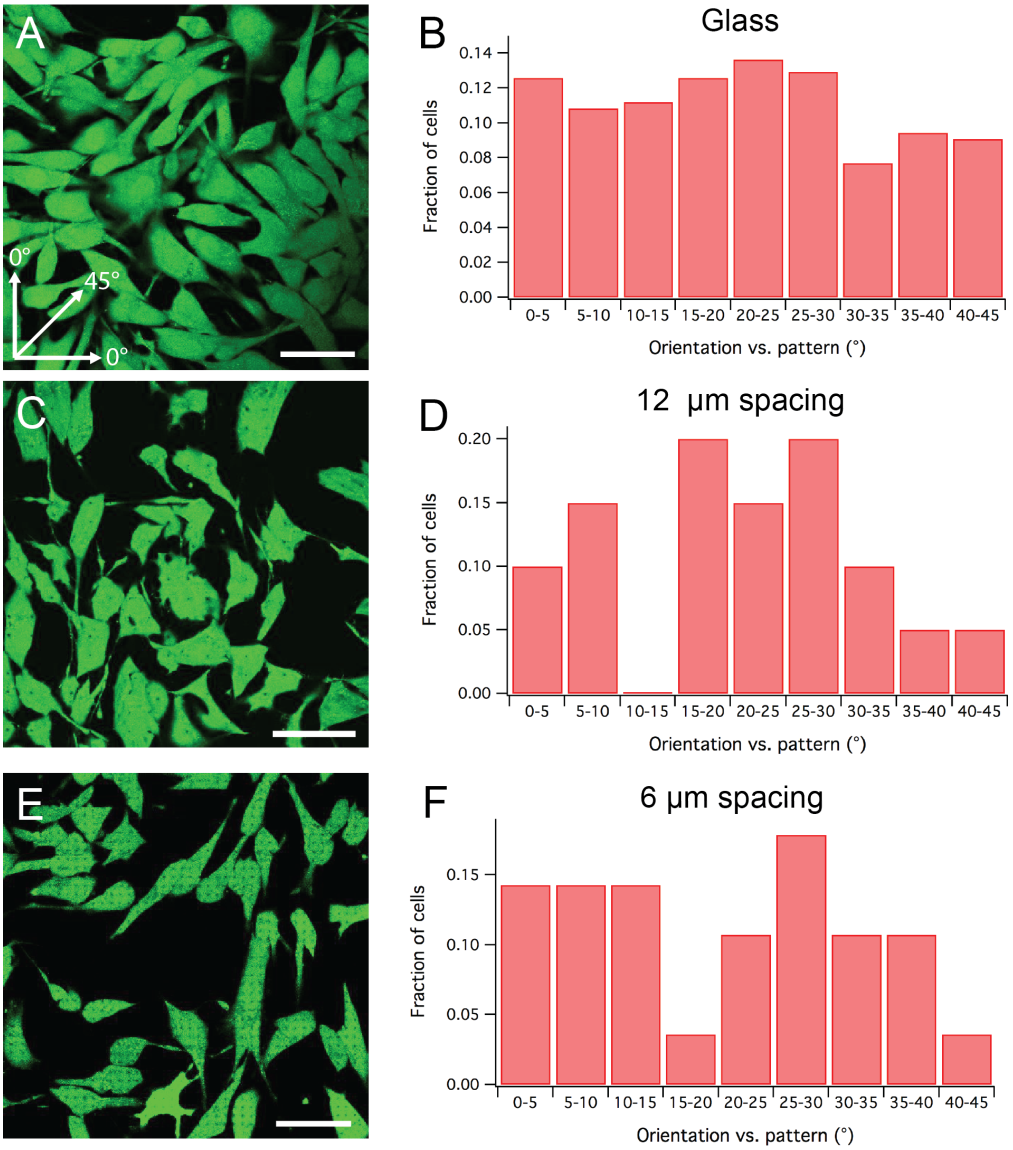

Figure S7-1. Confocal fluorescence overview images of calcein-AM stained NIH3T3 cells and the corresponding histograms of cell orientation on glass (A,B) or 3µm-long NPs spaced by 12 µm (C,D) or 6 µm (E,F). The orientation reference is shown in A and main text Figure 6B. The histogram data for each condition stem from at least two independent experiments with orientations quantified for ~60 cells in total. The scale bars in A,C and E represent 50 µm.

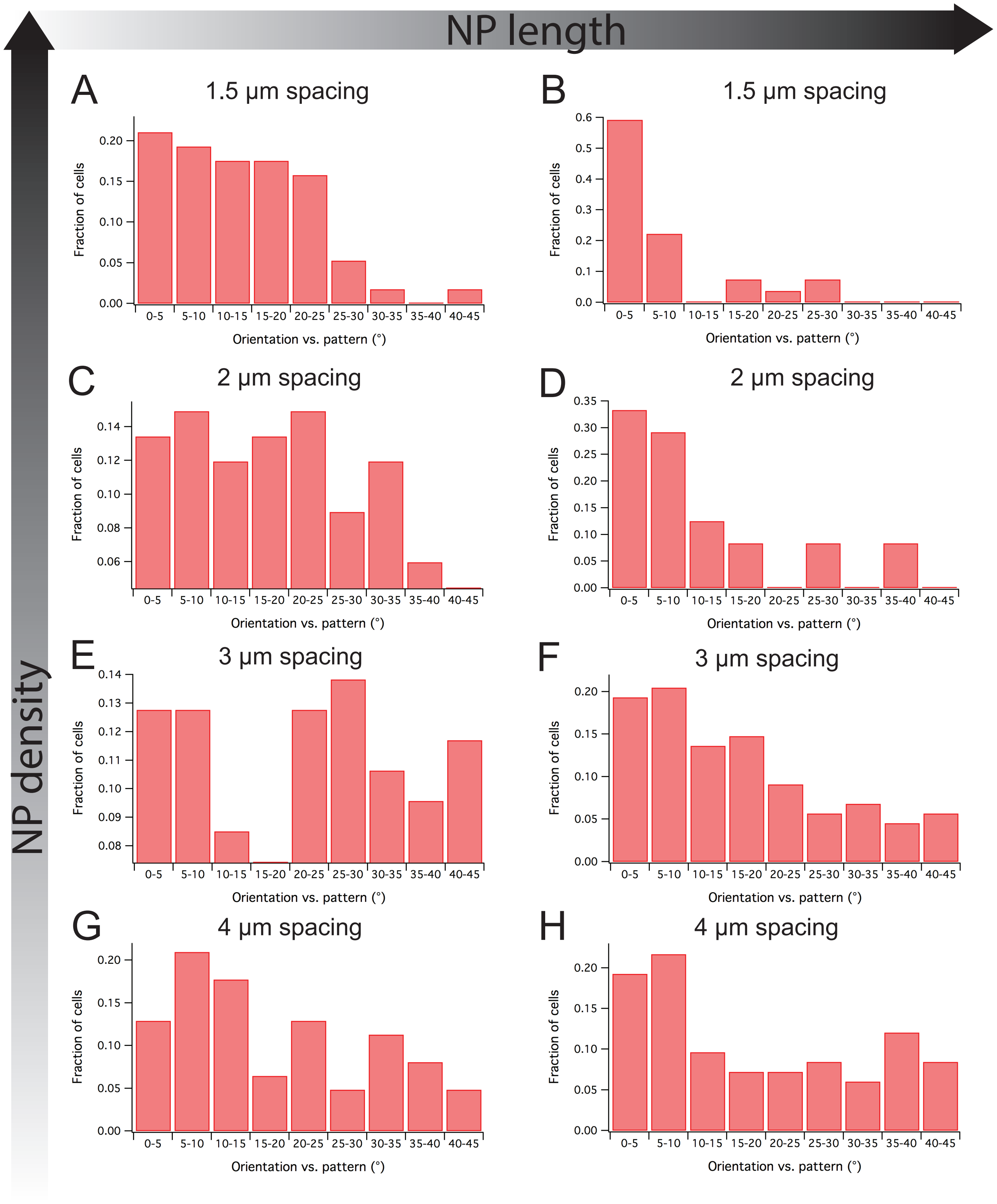

Figure S7-2. Histograms of NIH3T3 cell orientation (according to definition in Figure S7-1A and main text Figure 6B) on NPs with lengths 3 µm (A,C,E,G) or 6 µm (B,D,F,H) and spacings 1.5 (A,B), 2 (C,D), 3 (E,F) or 4 µm (G,H). The data for each condition stems from at least two independent experiments with orientations quantified for ~60 cells in total.

S8. Cell Alignment along NP Tips

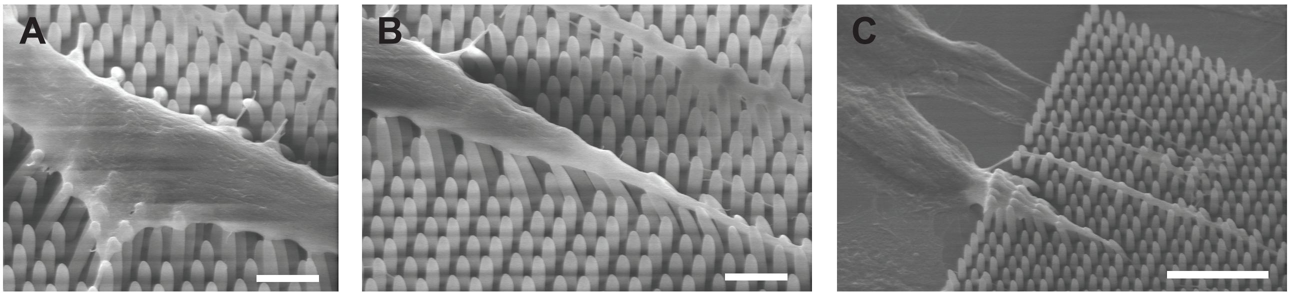

Figure S8. Cell alignment along NP tips. A,B) SEM images of the main cell body (A) and cell outgrowth (B) of the same cell on 1.5 µm NP spacing and 6 µm NP length. Scale bars represent 5 µm. C) SEM image at the border of the same NP array as in A,B, where the outgrowth of a cell adhering on the glass next to the NPs is seen to climb the NPs and continue along the tips of these. The scale bar represents 10 µm.

References

1 Kim, H. S. & Yoo, H. S. Differentiation and focal adhesion of adipose-derived stem cells on nano-pillars arrays with different spacing. *RSC Adv.* **5**, 49508-49512, doi:10.1039/C5RA07608K (2015).

2 van Hoorn, H. *et al.* The nanoscale architecture of force-bearing focal adhesions. *Nano Lett.* **14**, 4257-4262, doi:10.1021/nl5008773 (2014).

3 Kulangara, K. *et al.* The effect of substrate topography on direct reprogramming of fibroblasts to induced neurons. *Biomaterials* **35**, 5327-5336, doi:10.1016/j.biomaterials.2014.03.034 (2014).

4 Chen, W., Sun, Y. & Fu, J. Microfabricated Nanotopological Surfaces for Study of Adhesion-Dependent Cell Mechanosensitivity. *Small* **9**, 81-89, doi:10.1002/smll.201201098 (2013).

5 Sochol, R. D., Higa, A. T., Janairo, R. R. R., Li, S. & Lin, L. Unidirectional mechanical cellular stimuli via micropost array gradients. *Soft Matter* **7**, 4606-4609 (2011).

6 Ghibaudo, M., Di Meglio, J.-M., Hersen, P. & Ladoux, B. Mechanics of cell spreading within 3D-micropatterned environments. *Lab Chip* **11**, 805-812, doi:10.1039/C0LC00221F (2011).

7 Badique, F. *et al.* Directing nuclear deformation on micropillared surfaces by substrate geometry and cytoskeleton organization. *Biomaterials* **34**, 2991-3001, doi:10.1016/j.biomaterials.2013.01.018 (2013).

8 Unadkat, H. V. *et al.* An algorithm-based topographical biomaterials library to instruct cell fate. *Proc. Natl. Acad. Sci. USA* **108**, 16565-16570 (2011).

9 Davidson, P. M. *et al.* Topographically induced self-deformation of the nuclei of cells: dependence on cell type and proposed mechanisms. *J. Mater. Sci.: Mater. Med.* **21**, 939-946, doi:10.1007/s10856-009-3950-7 (2010).

10 Davidson, P. M., Özçelik, H., Hasirci, V., Reiter, G. & Anselme, K. Microstructured Surfaces Cause Severe but Non-Detrimental Deformation of the Cell Nucleus. *Adv. Mater.* **21**, 3586-3590, doi:10.1002/adma.200900582 (2009).

11 Pan, Z. *et al.* Control of cell nucleus shapes via micropillar patterns. *Biomaterials* **33**, 1730-1735, doi:10.1016/j.biomaterials.2011.11.023 (2012).
12 Ahn, E. H. *et al.* Spatial control of adult stem cell fate using nanotopographic cues. *Biomaterials* **35**, 2401-2410, doi:10.1016/j.biomaterials.2013.11.037 (2014).
13 Park, J. *et al.* Directed migration of cancer cells guided by the graded texture of the underlying matrix. *Nat. Mater.* **15**, 792-801, doi:10.1038/nmat4586 (2016).
14 Viela, F., Granados, D., Ayuso-Sacido, A. & Rodríguez, I. Biomechanical Cell Regulation by High Aspect Ratio Nanoimprinted Pillars. *Adv. Funct. Mater.* **26**, 5599-5609, doi:10.1002/adfm.201601817 (2016).
15 Cha, K. J., Hong, J. M., Cho, D.-W. & Kim, D. S. Enhanced osteogenic fate and function of MC3T3-E1 cells on nanoengineered polystyrene surfaces with nanopillar and nanopore arrays. *Biofabrication* **5**, 025007, doi:10.1088/1758-5082/5/2/025007 (2013).
16 Kuo, C.-W. *et al.* Polymeric nanopillar arrays for cell traction force measurements. *ELECTROPHORESIS* **31**, 3152-3158, doi:10.1002/elps.201000212 (2010).
17 Beckwith, K. S., Cooil, S. P., Wells, J. W. & Sikorski, P. Tunable high aspect ratio polymer nanostructures for cell interfaces. *Nanoscale* **7**, 8438-8450, doi:10.1039/c5nr00674k (2015).
18 Berthing, T. *et al.* Cell Membrane Conformation at Vertical Nanowire Array Interface Revealed by Fluorescence Imaging. *Nanotechnology* **23**, 415102, doi:10.1088/0957-4484/23/41/415102 (2012).
19 Buch-Månson, N. *et al.* Towards a Better Prediction of Cell Settling on Nanostructure Arrays-Simple Means to Complicated Ends. *Adv. Funct. Mater.* **25**, 3246-3255, doi:10.1002/adfm.201500399 (2015).
20 Kim, J.-H., Seo, S. & Min, J. Epithelial cell patterns on a PDMS polymer surface using a micro plasma structure. *J. Biotechnol.* **155**, 308-311, doi:10.1016/j.jbiotec.2011.07.009 (2011).
21 Ahn, J., Son, S. J. & Min, J. The control of cell adhesion on a PMMA polymer surface consisting of nanopillar arrays. *J. Biotechnol.* **164**, 543-548, doi:10.1016/j.jbiotec.2012.12.017 (2013).
